# Favorable outcomes following early onset oral miglustat in early infantile Niemann Pick Type C

**DOI:** 10.1016/j.ymgmr.2021.100739

**Published:** 2021-03-06

**Authors:** Shiri Curelaru, Yoav Zehavi, Tal Almagor, Ronen Spiegel

**Affiliations:** a Department of Pediatrics B, Emek Medical Center, Afula, Israel; b Rappaport School of Medicine, Technion, Haifa, Israel

**Keywords:** Niemann-Pick disease Type C, Lysosomal storage disease, *NPC1* gene, Miglustat, NPC, Niemann-Pick disease Type C, MRI, magnetic resonance imaging

## Abstract

Niemann-Pick disease Type C (NPC) is a rare autosomal recessive neurovisceral lysosomal disorder. Perinatal and early infantile onset NPC are the most severe types of the disease. Early infantile type is characterized by a rapidly progressive neurodegenerative course, which entails significant morbidity and usually results in death within 5 years. Miglustat, an iminosugar that selectively inhibits the glycosylceramide synthase enzyme, is known to stabilize or delay neurological progression in individuals with NPC, but its impact on affected infants is yet to be elucidated. We present two siblings with early infantile NPC due to the previously reported devastating homozygous mutation c.2279_2281delTCT in *NPC1*. Their considerably discrepant neurological disease courses were dependent on the timing of initiation of miglustat treatment. The outcomes support the significant role of early treatment with miglustat in the disease course of early infantile NPC and suggest that therapy should be considered even before the occurrence of neurological involvement. Moreover, this report emphasizes the importance of early diagnosis, in light of the availability of a potential disease-modifying medication.

## 1 Introduction

Niemann-Pick disease Type C (NPC) is a rare autosomal recessive neurovisceral lysosomal disorder currently estimated to occur in 1:100,000 to 1:120,000 live births [1]. The disease is caused by biallelic mutations in either the *NPC1* gene (in 95% of patients) or in the *NPC2* gene (in about 4% of patients) [2]. NPC is characterized by a heterogeneous clinical presentation, with a wide range of visceral, neurological, and psychiatric symptoms [1,2]. The disorder is further categorized by the age of onset of initial symptoms mainly neurological impairment. Accordingly, five disease groups are recognized: perinatal systemic (onset at age < 3 months), early-infantile (3 months to 2 years), late-infantile (2–6 years), juvenile (6–15 years), and adolescent/adult (> 15 years) [[1], [2], [3], [4]]. NPC disease presentation during the perinatal and early infantile periods may overlap; the most common presentation is prolonged neonatal jaundice (usually direct hyperbilirubinemia), with varying degrees of hepatosplenomegaly [1]. Neurological symptoms usually develop gradually with subtle signs. Truncal hypotonia generally occurs within the first year of life. Patients later begin to lose developmental milestones that were already gained; cerebellar ataxia and further motor deterioration are frequently observed within the second year of life [2,3]. Communication is relatively well maintained and is among the later skills to be lost. Vertical supranuclear gaze palsy, a common early manifestation in NPC, may present, but is difficult to recognize at an early age. Nevertheless, for a substantial group of neonates with the severe systemic perinatal NPC (8–10% of the patients), the hepatic manifestations may progress rapidly to fulminant liver disease or multi-organ failure, even before neurological symptoms appear, and subsequently lead to death within the first year of life [1,2]. Outcomes for patients with early-infantile/neonatal NPC are very poor. Neurological progression results in severe motor and cognitive impairment, quadriplegia, dystonia, convulsive disorder, and bulbar impairment, and consequently leads to dysphagia and chronic pulmonary disease due to recurrent aspirations; death often occurs within 5 years [2].

Miglustat (Zavesca) was initially approved in the European Union in 2009, for the treatment of adults and children with NPC [5,6]. Miglustat was the first and is currently the only targeted therapy approved for treating the neurological manifestation of NPC. Importantly, it has no effect on visceral such as splenomegaly or hepatic involvement and thus it has no effect on the severe perinatal systemic type. Several studies have shown favorable effects of miglustat on the natural disease course of NPC, mainly among adult patients [7,8]. Although its precise mechanism in NPC is not completely understood, the drug has beneficial effects on intracellular lipid trafficking. It reduces the apparently neurotoxic accumulation of glucosylceramide, lactosylceramide, and the gangliosides GM2 and GM3 in the brain; and delays the progression of neurological symptoms of NPC [9]. However, only limited data, mostly observational case descriptions, are available regarding its use in neonatal and early infantile onset NPC.

In this study we report the disease course in two brothers with a deleterious homozygous *NPC1* variant that was previously reported in patients with either the perinatal and early infantile presentation [10]. The older brother started treatment with miglustat at age 3 years, while already manifesting significantly progressive neurological disease. His younger brother started treatment at age 12 months, with subtle neurological signs. Our report provides further support for the beneficial effect of early administration of oral miglustat in the most severe forms of NPC.

## 2 Case reports

### 2.1 Patient 1

This patient is the older brother, the second child of healthy parents who are first degree cousins of Arab Muslim origin. His pregnancy was remarkable for intrauterine growth restriction. Fetal genetic studies were not performed, and fetal ultrasound examinations during the second and third trimesters of pregnancy were unremarkable. He was born at term, by normal vaginal delivery, with a low birth weight of 2250 g, and a proper perinatal course.

At age six weeks, the infant was investigated due to direct hyperbilirubinemia (total bilirubin 5 mg/dL, direct bilirubin 2 mg/dL). His weight and height at that time were both within the 5th centiles. His physical examination was normal except for hepatosplenomegaly. Serologies for common congenital infections and cytomegalovirus polymerase chain reaction (PCR) (tested from bloodspot obtained immediately after birth) were all negative. The brainstem evoked response audiometry test was intact. Complete blood count was normal. Serum biochemistry revealed elevated liver transaminases (aspartate transaminase 230 U/L [normal range: 0–35 U/L] and alanine transaminase 70 U/L [normal range: 0–45 U/L]). Alkaline phosphatase and gamma-glutamyl transferase were also elevated: 773 U/L [normal range: 70–155 U/L] and 173 U/L [normal ranges: 30–120 U/L], respectively. Abdominal ultrasound revealed an enlarged liver of 12 cm span and mild splenomegaly of 8 cm. Brain ultrasound was normal. Ophthalmological examination revealed posterior embryotoxon. Echocardiography was unremarkable, and butterfly vertebrae were evident on spinal radiography. A liver biopsy displayed nonspecific hepatitis with cholestasis and a moderate degree of hepatic fibrosis. Genetic analysis of the *JAG1* gene did not reveal a pathogenic variant. At age 6 months, spontaneous withdrawal of the cholestasis occurred, and the liver transaminases gradually decreased to normal levels. However, splenomegaly further progressed. His neurological development during the first year of life was relatively preserved. At age 6 months, he was able to flip over and grab toys with his hands. At age 10 months he managed to crawl. At age 18 months he had a vocabulary of about 10 words and was able to understand simple orders. He could sit straight when seated but was unable to sit by himself or stand with support. Brain magnetic resonance imaging (MRI) at age 18 months showed a relatively thin corpus callosum, mild ventriculomegaly, and an enlarged subarachnoid space.

The disease progressed notably during age 18–24 months, as developmental milestones already acquired were increasingly lost. Motor milestones were initially lost, followed by cognitive and communication skills. Hypertonia and quadriparesis gradually developed, in addition to generalized muscle atrophy, followed by truncal ataxia and intention tremor. He slowly developed dysphagia and coarse facial appearance. Skeletal involvement was marked by progressive chest deformation dominated by pectus carinatum, barrel chest, and progressive kyphotic deformities.

At age 3 years, the patient already had significant truncal hypotonia with four limb hypertonia and was unable to roll over or to grab an object. His upward gaze was limited, but he was able to track objects, smile, and recognize his parents; otherwise, he was unable to communicate.

Chromosomal microarray analysis revealed a normal male karyotype without copy number variations. Genetic studies were negative for the *SMPD1* gene; however, genetic analysis of NPC identified the homozygous c.2279_2281delTCT pathogenic variant in the *NPC1* gene, (p.Phe760del; NM_000271.5) which was previously reported in perinatal/neonatal patients with NPC [10].

Following the genetic diagnosis of NPC, the patient, aged 3.3 years, started therapy with miglustat 300 mg/day. However, the disease continued to progress, with further loss of developmental abilities as he became bedridden, quadriplegic, and unable to recognize his parents. Eventually, he lost all communication with his environment. The patient was unable to feed and required a gastrostomy tube. He suffered from pulmonary infections due to recurrent aspirations and died at age 6 years.

### 2.2 Patient 2

This is the younger brother of patient 1. During the last trimester of pregnancy, fetal ultrasound showed mild hepatomegaly; a subsequent fetal MRI scan at 33 weeks gestation was normal. Fetal chromosomal microarray analysis revealed a male karyotype with otherwise no copy number variations. He was born vaginally at 37 weeks gestation with a birth weight of 2600 g.

Growth and development were normal within the first six months of life but thereafter the patient displayed mild truncal hypotonia characterized by late attainment of motor milestones. In addition, his physical examination revealed asymptomatic splenomegaly. At age 10 months, his older brother (patient 1) was diagnosed genetically with NPC. Given his splenomegaly and hypotonia, patient 2 was tested for the c.2279_2281delTCT *NPC1* variant and was found to be homozygous. Given the early diagnosis, the severe early phenotype associated with this specific mutation, and the patient's mild neurological impairment, he was started with miglustat therapy (250 mg/m^2^ daily) at age 12 months.

Following initiation of miglustat treatment, the patient made slow but steady developmental progress. At age 20 months, he was able to sit by himself and utter several words. At age 21 months, he was able to stand and walk with support, and at 30 months his vocabulary was age appropriate. However, he showed further systemic manifestations including coarseness of his face, upper airway obstruction due to adenoid hypertrophy, a barrel-shaped chest, and mild kyphosis. Abdominal ultrasound revealed a significantly increased spleen with 14 cm span. His brain MRI at 2.5 years of age was normal.

The patient has since made steady neurological progress and continues to gain development milestones. His miglustat dosage was gradually increased to 300 mg daily, without side effects. On his last examination at 3.5 years he was able to stand and walk with support, and to speak short sentences composed of several words; his cognitive development is age appropriate. However, he has significant fine motor impairment, as well as dysmetria and intention tremor. He also has mild pyramidal signs with exaggerated deep patellar tendon reflexes (+3) on both sides, without clonus, and a negative Babinski sign. A repeated brain MRI scan, at age 3.5 years, was normal and audiologic assessment showed mild conductive hearing impairment of 20-25 dB decline.

## 3 Discussion

This report presents a considerably discordant disease course in two siblings with early infantile NPC who initiated miglustat treatment at very different stages. The homozygous pathogenic variant c.2279_2281delTCT (p.Phe760del) shared by the brothers was previously reported by our group in three individuals who presented with the severe perinatal form (2 patients) and the early infantile form (1 patient) of NPC [10]. Accordingly, the three previously reported patients had a grave course; two of them developed end-stage liver disease and died before age 6 months. The third patient developed rapid neurological deterioration around age 12 months and early lethality at age 3.5 years, when already bedridden and non-communicative [10]. An additional patient, with the same homozygous mutation has had a similar early infantile disease course and is currently in a vegetative state at age 5 years (personal communication). Notably, this patient also started miglustat treatment at age three years, when the drug was initially approved and introduced into the Israeli national health basket. Nevertheless, at the initiation of miglustat therapy, the patient already had severe global neurological impairment, and he continued to deteriorate neurologically despite the treatment. The clinical features and disease course of all six patients with similar *NPC1* genotype are summarized in Table 1. Correspondingly, patients homozygous to the devastating c.2279_2281delTCT *NPC1* variant are destined to either the severe lethal perinatal form or early infantile disease subtype, with severe course, rapidly progressive neurodegeneration, and early lethality during the first years of life. This discordance between siblings or individuals sharing similar *NPC1* genotypes who may present with variable clinical phenotype (perinatal and early infantile) is already documented in NPC [3]. When miglustat was initiated to patients aged three years, with rapid deterioration (patients 1 and 6, Table 1), no beneficial effect on disease progression was observed. However, when therapy was started much earlier, at age one year, the disease course was considerably favorable.

**Table 1:** Clinical manifestations of patients with *NPC1* gene homozygous pathogenic variant c.2279_2281delTCT (p.Phe760del).

	Patient 1 (older brother)	Patient 2 (younger brother)	Patient 3	Patient 4	Patient 5	Patient 6
Gender	Male	Male	Female	Male	Female	Male
Current age (or age at death)	Died at 6Y	3.5Y	Died at 5Mo	Died at 2Mo	Died at 3.5Y	5Y
Prenatal manifestations	IUGR	Hepatomegaly	Hepatosplenomegaly, IUGR, oligohydramnios	Hepatosplenomegaly, fetal ascites, prematurity of 31 weeks	IUGR	Fetal ascites
Postnatal manifestations						
Hepatosplenomegaly (age of diagnosis)	Yes (6 weeks)	Yes (prenatally)	Yes (prenatally)	Yes (prenatally)	Yes (infancy)	Yes (neonatally)
Cholestatic jaundice	Yes	No	Yes	Yes	No	Yes
Hepatic failure	No	No	Fulminant	Fulminant	No	Severe with recovery
Congenital thrombocytopenia	No	No	yes	yes	yes	yes
Skeletal features	Coarse face, pectus carinatum and barrel chest, kyphotic deformation	Coarse face, barrel chest, kyphotic deformation	No	No	Coarse face, barrel chest, kyphotic deformation	Coarse face, pectus carinatum and barrel chest, kyphotic deformation
Neurological manifestations						
Hypotonia (age at diagnosis)	Yes (12Mo)	Yes (10Mo)	No	No	Yes (8Mo)	Yes (10Mo)
Dysmetria	Yes	Yes	No	No	Yes	Yes
Convulsive disorder (age at onset)	Yes (2.5Y)	No	No	No	Yes (2Y)	Yes (3Y)
Best motor milestone achieved (age)	Assisted sitting (15Mo)	Assisted walking (21Mo)	Head control	Head control	Assisted standing (12Mo)	Assisted sitting (15Mo)
Best cognitive milestone achieved (age)	~10 spoken words (15 Mo)	Appropriate speech (36Mo)	social smile	Eye contact	2–3 words (12Mo)	Two words (15Mo)
Age of initial loss of already achieved milestones	18Mo	3Y	No loss	No loss	12Mo	18Mo
Gastrostomy tube insertion (age)	Yes (24Mo)	No	No	No	Yes (30Mo)	Yes (3Y)
Oxygen supplementation	Yes	No	No	No	Yes	Yes
Hearing impairment	Mild	Mild	Not evaluated	Not evaluated	Moderate	Moderate
Current neurology condition (or before death)	Vegetative	Moderate hypotonia, dysmetria	Hypotonia	Hypotonia	Vegetative	Vegetative
Brain MRI (age, findings)	Yes (18 Mo, thin corpus callosum, mild ventriculomegaly and enlarged subarachnoid space)	Yes (Intrauterine, normal; 2.5Y and 3.5Y, normal)	Not performed	Not performed	Yes (18Mo, delayed maturation of white matter; 36Mo, severe cortical atrophy, thinning of corpus callosum and demyelination)	Not performed
Miglustat treatment (age of beginning)	Yes (3.3Y)	Yes (12Mo)	No	No	No	Yes (3Y)
Disease subtype	Early infantile	Early infantile	Severe perinatal	Severe perinatal	Early infantile	Early infantile
Reference	Current study	Current study	Spiegel et al.^9^	Spiegel et al.^9^	Spiegel et al.^9^	Personal communication

Abbreviations in Table 1: Mo = months, Y = years, IUGR = Intrauterine growth restriction, MRI = Magnetic resonance imaging.

Patients 3-5 were previously described [10].

Our study corroborates previous reports of pediatric French and Spanish NPC cohorts treated with miglustat. Specifically, further deterioration of the neurological disease course was demonstrated in the majority of early infantile patients, compared to higher rates of disease stabilization or even improvement among late infantile and juvenile patients [11,12]. In agreement, most of the early infantile patients reported in these two cohorts started treatment after age two years, presumably when the disease was already significantly progressive. Remarkably, one of the patients in the early infantile French cohort started treatment at age 9 months and displayed consistent improvement during 15 months of therapy but began to deteriorate progressively thereafter [11].

Traditionally, miglustat treatment is recommended for treatment of NPC at the earliest sign of neurological impairment in all age groups [7,8]. However, data are currently limited regarding the effect of very early treatment with miglustat in either neonatal or early infantile onset NPC. Given the rarity of the early infantile subgroup, well-designed controlled studies are almost impractical and our knowledge of the effect of the drug on disease progression relies mainly on observational case descriptions. Most patients with early onset NPC who were treated with miglustat, started their treatment relatively late, when the disease course was already progressive, and the benefit of treatment was therefore substantially limited [11,12,14]. Only few case reports have described miglustat therapy before age one year in patients with early onset NPC [13,15,16]. In one of these, treatment was initiated very early, at age 4 months, in a patient with early infantile NPC (compound heterozygous *NPC1* mutations) who already displayed hypotonia and developmental delay. This patient made neurological progress and was able to walk independently at age 24 months. [13]. Based on these sporadic yet encouraging reports, early preventive treatment with miglustat was intuitively suggested for patients with early onset NPC, even before neurological symptoms appear. Still, in most of these patients, genotypes are private and unique, and therefore the disease course, mainly the neurological progression, is virtually unpredicted. An additional report described a patient with late infantile onset NPC, the brother of an older sister who already manifested neurological impairment and in whom treatment was started at the age of 16 months before neurological impairment occurred. In this same report the authors describe another female with early infantile onset NPC who started miglustat as early as 7 months. In these two patients, early treatment seemed to demonstrate a clear beneficial effect [16]. However, a follow-up report three years later documented progressive neurological impairment despite the therapy [17] suggesting miglustat therapy in these patients may delay or slow the progression of neurological disease but has a limited long-term effect.

Our additional three patients with early infantile onset NPC, with the same homozygous mutation, can be considered a late/non-intervention control group for early miglustat treatment in patient 2. The latter started treatment relatively early, before the disease became rapidly progressive. This contrasts with the previous reports of early miglustat therapy that were unable to compare with the natural course of disease. Our findings support the previously suggested hypothesis that miglustat treatment should be started as early as possible and even before evidence of neurological impairment, preferably immediately after genetic diagnosis, especially in early onset disease. Despite initiation of miglustat treatment at around age 3 years, two of our patients suffered a devastating disease course. This indicates a narrow window of opportunity for successful outcome and emphasizes the importance of early diagnosis. Thus, in very early onset disease forms of NPC (infantile onset), treatment should be started early, even before the appearance of neurological symptoms that signify irreversible damage of the central nervous system. This contrasts with late onset NPC (juvenile and adult forms), in which miglustat is still effective even in later disease stages. Finally, we further highlight the importance of high suspicion of NPC in infants with initial signs of splenomegaly and direct hyperbilirubinemia, either with or without hepatic impairment, given the availability of effective early treatment.

## 4 Conclusions

This report confirms the role of miglustat as a disease modifying treatment in neonatal and early infantile NPC only when started very early in the course of the disease. Future studies of additional patients with infantile NPC, with longer follow-up periods, are needed to better define the long-term therapeutic effect of miglustat.

## Ethical approval

All procedures performed in the study were in accordance with the ethical standards of the institutional research committee and with the 1964 Helsinki declaration and its later amendments or comparable ethical standards.

## Consent for publication

Participant gave consent for publication. The data of patients included in the study are unidentified.

## Authors' contributions

Dr. Curelaro performed the data analysis and investigation and led the medical writing.

Dr. Zehavi participated in the formal analysis, investigation and data curation and reviewed and edited the manuscript.

Dr. Almagor participated in the formal analysis, investigation and reviewed and edited the manuscript.

Prof. Spiegel designed conceptualized and supervised the study and led the medical writing

## Funding

This research did not receive any specific grant from funding agencies in the public, commercial, or not-for-profit sectors.

## Declaration of Competing Interest

All the authors declare that they have no conflict of interest.
